# Systematic review of quality of life and functional outcomes in randomized placebo-controlled studies of medications for attention-deficit/hyperactivity disorder

**DOI:** 10.1007/s00787-017-0986-y

**Published:** 2017-04-20

**Authors:** David R. Coghill, Tobias Banaschewski, César Soutullo, Matthew G. Cottingham, Alessandro Zuddas

**Affiliations:** 1 Department of Paediatrics, Faculty of Medicine, Dentistry and Health Science, University of Melbourne, Melbourne, VIC Australia; 2 Department of Psychiatry, Faculty of Medicine, Dentistry and Health Science, University of Melbourne, Melbourne, VIC Australia; 3 Division of Neuroscience, University of Dundee, Dundee, UK; 4 Department of Child and Adolescent Psychiatry and Psychotherapy, Central Institute of Mental Health, Medical Faculty Mannheim, University of Heidelberg, Mannheim, Germany; 5 Child and Adolescent Psychiatry Unit, Department of Psychiatry and Medical Psychology, University of Navarra Clinic, Pamplona, Spain; 6 Oxford PharmaGenesis, Oxford, UK; 7 Child and Adolescent Psychiatry Unit, Department of Biomedical Sciences, University of Cagliari, Cagliari, Italy

**Keywords:** Attention-deficit/hyperactivity disorder, Quality of life, Functional impairment, Randomized clinical trials, Systematic review

## Abstract

Children, adolescents and adults with attention-deficit/hyperactivity disorder (ADHD) experience functional impairment and poor health-related quality of life (HRQoL) in addition to symptoms of inattention/hyperactivity–impulsivity. To synthesize qualitatively the published evidence from randomized, double-blind, placebo-controlled trials of the effectiveness of pharmacotherapy on functional impairment or HRQoL in patients with ADHD, a systematic PubMed searching and screening strategy was designed to identify journal articles meeting pre-specified criteria. Post hoc analyses and meta-analyses were excluded. HRQoL outcomes, functional outcomes and the principal ADHD symptom-based outcome were extracted from included studies. An effect size of 0.5 versus placebo was used as a threshold for potential clinical relevance (unreported effect sizes were calculated when possible). Of 291 records screened, 35 articles describing 34 studies were included. HRQoL/functioning was usually self-rated in adults and proxy-rated in children/adolescents. Baseline data indicated substantial HRQoL deficits in children/adolescents. Placebo-adjusted effects of medication on ADHD symptoms, HRQoL and functioning, respectively, were statistically or nominally significant in 18/18, 10/12 and 7/9 studies in children/adolescents and 14/16, 9/11 and 9/10 studies in adults. Effect sizes were ≥0.5 versus placebo for symptoms, HRQoL and functioning, respectively, in 14/16, 7/9 and 4/8 studies in children/adolescents; and 6/12, 1/6 and 1/8 studies in adults. Effect sizes were typically larger for stimulants than for non-stimulants, for symptoms than for HRQoL/functioning, and for children/adolescents than for adults. The efficacy of ADHD medication extends beyond symptom control and may help reduce the related but distinct functional impairments and HRQoL deficits in patients with ADHD.

**Electronic supplementary material:**

The online version of this article (doi:10.1007/s00787-017-0986-y) contains supplementary material, which is available to authorized users.

## Introduction

Attention-deficit/hyperactivity disorder (ADHD) affects approximately 6.8% of children, 2.8% of adolescents and 2.5% of adults worldwide [75, 76, 83]. The disorder is defined as persistent and developmentally inappropriate symptoms of inattention and/or hyperactivity–impulsivity that interfere with a patient’s social, academic and/or occupational functioning [8, 9]. The nature of functional impairment varies from patient to patient and with age and may encompass diverse outcomes such as underperformance at school or at work, unemployment or low income, substance abuse, smoking, teenage pregnancy, arrest, divorce or acquiring sexually transmitted disease [61]. Health-related quality of life (HRQoL) is generally acknowledged to represent the impact of ill-health on an individual’s ‘perception of their position in life, in the context of culture and value systems in which they live, and in relation to their goals, expectations, standards and concerns’ [2, 104]. It has become clear that ADHD has a negative impact on patients’ HRQoL and that this may be further exacerbated by, or may increase the risk of, other psychiatric conditions such as anxiety and depression [31, 34, 54, 93]. Impaired day-to-day functioning in domains such as educational achievement and interpersonal relations is the reason that most often underlies a patient’s or parent’s decision to seek medical advice [72].

Pharmacotherapies for ADHD include the psychostimulants methylphenidate (MPH) and amphetamines [including the prodrug lisdexamfetamine (LDX)], and the non-stimulants atomoxetine (ATX) and guanfacine. Guidelines recommend that drug therapy is used as part of a multi-modal treatment plan, which should include psychoeducation or other non-pharmacological interventions such as parent training and cognitive behavioural therapy [13, 102]. Randomized controlled trials of pharmacotherapies in children, adolescents and adults with ADHD have typically used a clinician-rated symptom scale as the primary efficacy outcome measure, and meta-analyses of these studies have confirmed that ADHD medications are very effective in relieving patients’ symptoms [33, 77, 88, 89]. As recognized in the recent European Medicines Agency guidance on inclusion of functional outcomes in clinical studies of ADHD medications [44], it is now widely acknowledged that treatment of ADHD should aim not only to relieve patients’ symptoms, but also to improve their functioning and HRQoL.

**Fig. 1:**
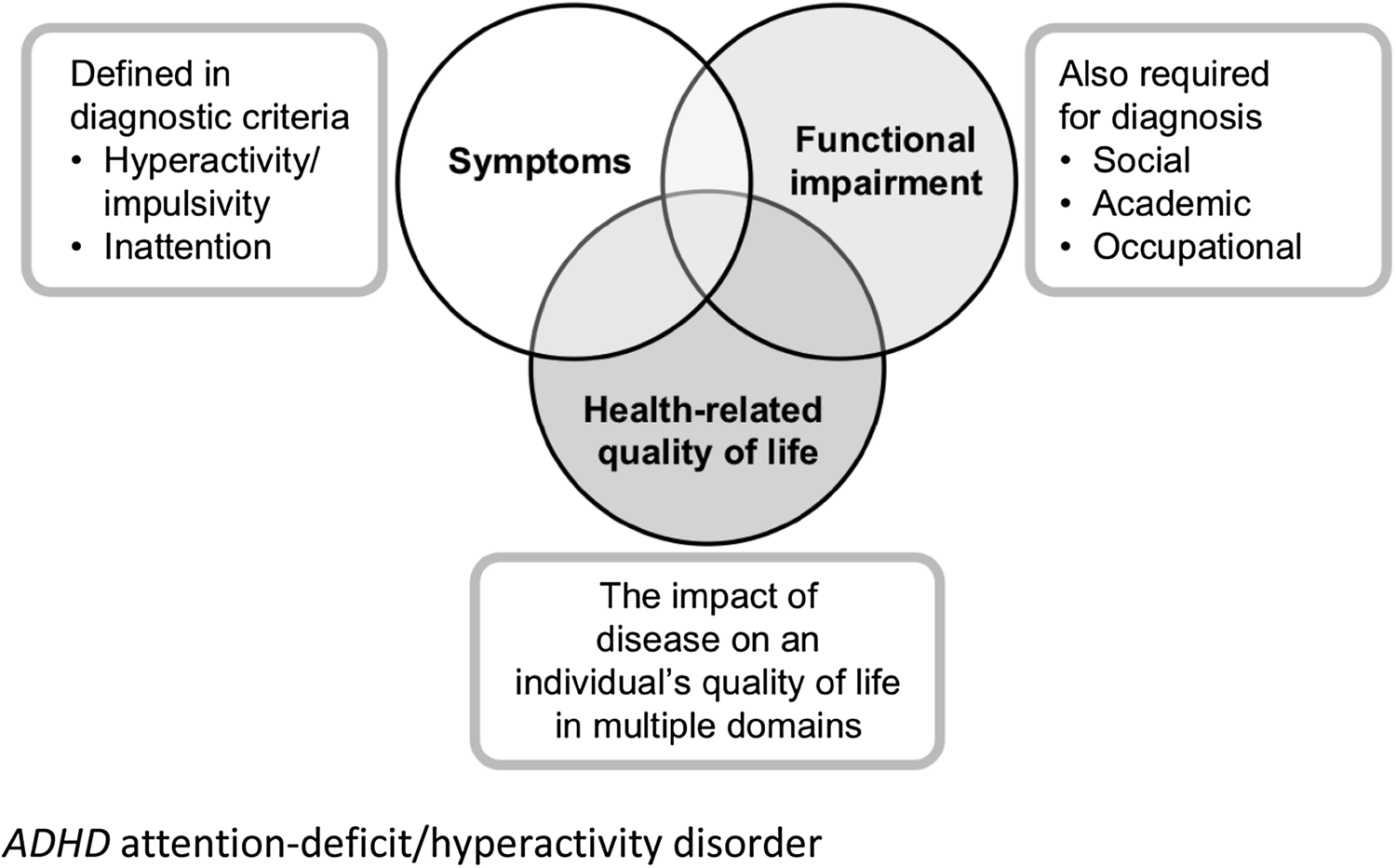
Effects of ADHD on a patient’s life extend beyond symptoms. *ADHD* attention-deficit/hyperactivity disorder

Assessing functional impairment or HRQoL in a randomized controlled trial typically relies on completion of one or more of the many available ADHD-specific or generic questionnaires by patients themselves or by proxy raters (physicians, parents, teachers or family members) [30]. Use of these measures in clinical trials of ADHD medications has grown rapidly in recent years [30]. Because ADHD is a behavioural disorder that affects multiple aspects of patients’ lives, the 18 well-defined symptoms of ADHD interact and partially intersect with the constructs of functional impairment and HRQoL (Fig. 1). Although measures of functional impairment may share many similarities with measures of HRQoL, functional impairment is usually considered to be objective and ideally assessed by unbiased methods, whereas HRQoL is usually considered to be subjective and ideally assessed by the patients themselves. What precisely is being measured by a patient-rated functional impairment instrument or a proxy-rated HRQoL instrument may therefore be open to debate and discussion [2]. Another important aspect of measuring functional impairment and HRQoL is that the instruments should sample domains of impairment that are commonly affected by patients’ symptoms, but should not merely serve as surrogate measures of symptoms. Moderate, but not strong, correlations between measures of symptoms, functional impairment and HRQoL suggest that related, but distinct, constructs are being assessed [18, 19, 21, 32, 47, 81, 92, 95].

These factors present challenges for the development and selection of instruments to measure HRQoL and functional impairment. Generic instruments have the advantage of capturing a broad picture of health status, but the potential disadvantage of poor sensitivity to the deficits characteristic of ADHD. Disorder-specific instruments may offer improved sensitivity by specifying particular impairments, but may miss others. When any single patient typically reports impairment in only a small proportion of the specified items within a domain, the instrument may suffer from poor sensitivity if the scoring system averages out this potentially severe and clinically relevant impairment across all the specified items within that domain. US Food and Drug Administration guidance on patient-reported outcome measures states that instruments should be psychometrically validated and standardized to a reference population [46]. Standardization may present problems for ADHD-specific instruments if they are not designed for use in people who do not have ADHD. An advantage of generic instruments that are standardized to a reference population is that they enable direct comparison of the burden of disease in patients with ADHD with that of community norms and patients with other physical or mental health problems.

Results from clinical trials and observational studies using questionnaire-based instruments indicate that patients with ADHD experience severely compromised HRQoL [34]. In a 2010 systematic review of clinical trials, open-label studies and post hoc analyses, evidence supporting a positive short-term effect of medication on quality of life was limited mainly to studies of ATX in children and adolescents [29]. Recent evidence from observational and registry studies indicates that pharmacological treatment of ADHD is associated with increased achievement and decreased absenteeism at school [16], a reduced risk of trauma-related emergency hospital visits [58], reduced risks of suicide and attempted suicide [28], and decreased rates of substance abuse [27] and criminality [56].

This systematic review focuses on the effect of pharmacological treatment of ADHD on functional impairment and HRQoL outcomes in randomized placebo-controlled studies. We aim to: survey the instruments used in these studies; assess the severity of baseline impairment relative to controls, when possible; collate the most robust evidence about the impact of medications on HRQoL and functional impairment outcomes; and assess the relationship of treatment-related changes in these measures with changes in ADHD symptom scales.

## Methods

A systematic literature searching and screening strategy was designed to identify articles published in peer-reviewed journals that reported findings of randomized placebo-controlled clinical trials of pharmacotherapy for ADHD using measures of functional impairment or HRQoL as outcomes. We extracted these outcomes for qualitative review and analysis, together with the principal ADHD symptom-based outcomes from the same studies.

This systematic review was conducted in accordance with the Preferred Reporting Items for Systematic Reviews and Meta-Analyses (PRISMA) guidelines and was registered with the PROSPERO international prospective register of systematic reviews (identifier: CRD42015027595).

### Search and selection

PubMed (http://www.ncbi.nlm.nih.gov/pubmed) was searched on 29 June 2016 using a search string (see Supplementary Table 1) designed to identify journal articles in English that reported the results of randomized, placebo-controlled, human clinical trials of pharmacological medications for ADHD that included assessments of functional impairment or HRQoL as study outcomes. Two individuals screened records independently and resolved all disagreements by discussion. In a first screen based on title and abstract, articles were excluded if they clearly failed to meet the inclusion criteria or met any of the exclusion criteria. In a second screen based on full text, articles were included only if they met all inclusion criteria and no exclusion criterion. References cited in all included articles were then screened in the same way.

### Inclusion and exclusion criteria

Studies were included only if they enrolled participants with a primary diagnosis of ADHD. Participants could be children, adolescents and/or adults. ADHD diagnosis had to be based on the Diagnostic and Statistical Manual of Mental Disorders (DSM) III, IIIR, IV, IV-TR or 5 criteria [8–12]; the International Statistical Classification of Diseases 10th Revision (ICD-10) criteria [103]; or on a diagnostic instrument that uses these criteria (e.g. ADHD Rating Scale IV [ADHD-RS-IV] [39], Schedule for Affective Disorders and Schizophrenia for School-Age Children-Present and Lifetime Version (K-SADS-PL) [53], or Conners’ Adult ADHD Diagnostic Interview for DSM-IV [CAADID] [41]). Studies in patients with a primary diagnosis of another mental disorder and secondary comorbid ADHD were excluded.

Studies were included only if they assessed the effects of medication alone or in combination with non-pharmacological interventions. Medications did not need to have regulatory approval for the treatment of ADHD. Folic acid, omega-3 fatty acids and other dietary supplements were not considered to be medications.

Only randomized placebo-controlled studies with prospectively defined comparisons between medication(s) and placebo were included. Studies could have parallel-group, crossover, treatment initiation or treatment withdrawal designs. Studies with an active control were included only if they also had a placebo control or placebo reference arm. Reviews and articles reporting post hoc analyses and meta-analyses were excluded.

Only studies that assessed patients’ functional impairment (or functioning) and/or HRQoL [or quality of life (QoL)] during randomized placebo-controlled treatment (not in a separate uncontrolled study period) were included. Instruments used for assessing these outcomes could be generic or disease-specific; investigator-rated, proxy-rated (e.g. by parents or teachers) or patient-rated; and did not need to be psychometrically validated. Assessments of any of the following were not eligible as measures of functional impairment: executive function, emotional dysregulation, emotional lability, mood, behaviour, (neuro)cognition, (neuro)cognitive function, (neuro)development, neuropsychiatric function, memory, reaction time, alertness, psychomotor function, response inhibition, pre-pulse inhibition, lexical function, intelligence, sleep, creativity, anxiety and depression. ‘Global functioning’ measured using the Clinical Global Impressions (CGI) scale was also not considered to be an eligible functional measure.

### Data extraction and analysis

The following study details were extracted from each included article: duration of the randomized assessment period, age range of participants, active treatment(s) and doses, number of participants and randomization ratio. For each study, effect sizes and *p* values were extracted for functional impairment outcomes, HRQoL outcomes and the principal symptom-based outcome (e.g. ADHD-RS-IV). When necessary, data for symptom-based outcomes of a study were extracted from references cited in the included article. If relevant results from a single study were published in more than one article, the articles were treated as a single entity. When effect sizes were not reported, they were calculated from published data if possible (using ‘*n*’, mean change and standard deviation or standard error of the mean; or ‘*n*’ and F-statistic). An effect size threshold of 0.5 was used as an indicator of minimum clinically important differences in the qualitative analysis [71].

The registered protocol for this systematic review (PROSPERO identifier: CRD42015027595) stated that meta-analysis would not be conducted unless sufficient studies of the same medication using the same functional/(HR)QoL outcome measure over approximately the same treatment period were considered to have been identified in the searches.

### Risk of bias

This review includes only study outcomes published in peer-reviewed journals. Functional impairment and HRQoL are usually secondary efficacy outcomes in ADHD studies and may be less likely to be reported than primary efficacy outcomes (e.g. ADHD symptoms). This could lead to bias if studies or study outcomes that indicate favourable effects of medication are more likely to be published that those that do not. We did not formally assess the risk of bias in the studies or outcomes included in this review.

Post hoc analyses were excluded because they may be more likely than pre-specified analyses to report favourable effects of medication and are not typically controlled for multiple statistical comparisons. Pre-specified analyses of secondary efficacy outcomes, however, may also not have been controlled for multiplicity of comparison and may be more likely to yield significant *p* values than primary analyses or secondary analyses that were included in a study-wide algorithm to control type I error. The *p* values quoted from some included studies may therefore represent nominal rather than strict statistical significance; both nominally significant and statistically significant *p* values are described as ‘significant’ in this review. To mitigate the associated risk of bias in favour of medication, we focus, when possible, on effect sizes of active medication versus placebo rather than *p* values to assess the efficacy of ADHD pharmacotherapy. This approach could lead to bias if studies with published or calculable effect sizes are more likely than those without available effect sizes to report favourable effects of medication.

## Results

### Study selection

**Fig. 2:**
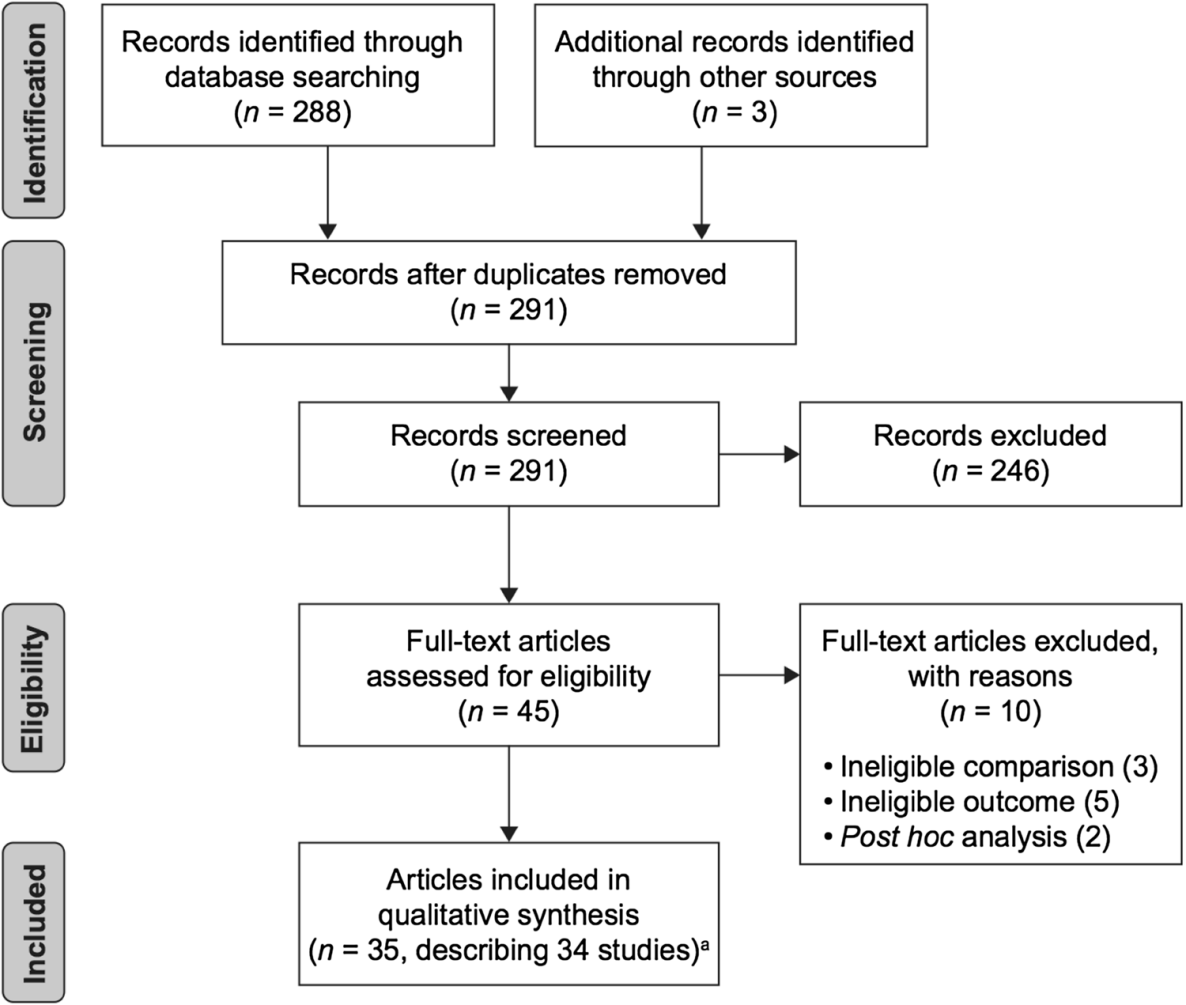
PRISMA diagram. ^a^35 articles describing 34 studies. Symptom-based outcome data for studies reported in eight included articles were extracted from an additional eight articles cited in the included articles. *PRISMA* Preferred Reporting Items for Systematic Reviews and Meta-Analyses

Of 288 articles identified by the search, 244 were excluded during screening of the title and abstract and a further ten were excluded during full-text screening. Articles excluded at full-text screening reported studies that: lacked a comparison of medication with placebo [20, 60, 87], lacked an eligible HRQoL or functional impairment outcome [23, 24, 26, 63, 74] or were post hoc analyses [62, 69]. The references cited in the remaining 34 included articles and in previous systematic reviews [29, 34, 88] were inspected, yielding three additional articles for screening based on title and abstract. Of these, two were excluded and one was screened based on full text and included [68], bringing the total number of included articles to 35. Two of these reported HRQoL data from the same study [84, 85], meaning that 34 studies were included for qualitative analysis (Fig. 2). Study data were extracted from all of the included articles and from eight additional articles that reported the symptom-based outcomes for the included studies (Tables 1, 2). A meta-analysis was not conducted because the included articles were not considered to report enough comparable data from studies of the same medications in similar populations and of similar duration for quantitative synthesis.

**Table 1:** Overview of HRQoL and functional outcomes from included randomized, placebo-controlled studies of medications in children and/or adolescents with ADHD

References	Duration of randomized assessment period	Age, years	Active treatment group(s)	Number randomized (ratio, active[s]: placebo)	Principal symptom-based efficacy outcome versus placebo at study endpoint or last assessment	HRQoL/functional outcomes: significant effect(s) of medication(s) versus placebo at study endpoint or last assessment	HRQoL/functional outcomes: other reported comparisons of medication(s) versus placebo
Short-term studies—stimulant medications							
Banaschewski et al. 2013 [15]	7 weeks	6–17	LDX (30, 50 or 70 mg/day, optimized); OROS-MPH reference (18, 36 or 54 mg/day, optimized)	336 (1:1:1)	ADHD-RS-IV total score (effect sizes LDX, 1.80***; OROS-MPH, 1.26***)	CHIP-CE:PRF^a^ Achievement (effect sizes LDX, 1.280***; OROS-MPH, 0.912***), Risk Avoidance (1.079***; 0.948***), Resilience (0.421**; 0.398*), Satisfaction (0.365*; 0.349*) WFIRS-P total score (effect size LDX, 0.924***; OROS-MPH, 0.772***), Family (0.730***; 0.646***), Learning and School (1.249***; 0.910***), Social Activities (0.643***; 0.642***), Risky Activities (0.640***; 0.414*), Life Skills (OROS-MPH, 0.348*), Child’s Self-Concept (OROS-MPH, 0.362*)	CHIP-CE:PRF^a^ Comfort (effect sizes LDX, 0.003^NS^; OROS-MPH, 0.181^NS^) WFIRS-P Life Skills (effect size LDX, 0.241^NS^), Child’s Self-Concept (LDX, 0.264^NS^)
Findling et al. 2011 [45]	4 weeks	13–17	LDX (30, 50 or 70 mg/day, forced titration)	314 (1:1:1:1)	ADHD-RS-IV total score (LDX 30 mg**, 50 mg**, 70 mg**)	None	YQOL-R overall score^NS^, Sense of Self^NS^, Social Relationships^NS^, Environment^NS^, General QoL^NS^, contextual items^NS^, item 57^NS^
Greenhill et al. 2006 [50]	7 weeks	6–17	*d*-MPH-ER (5, 10, 15, 20, or 30 mg/day, optimized)	103 (1:1)	CADS-T total score (effect size 0.79***)	CHQ-PF50^a^ Psychosocial summary***	CHQ-PF50^a^ Physical summary^NS^
Wilens et al. 2010 [99]	2 weeks	6–12	TD-MPH (20 mg, forced titration)	30 (1:1 crossover)	ADHD-RS-IV total score***	Investigator-rated BSFQ^a^ total score**	Patient-rated BSFQ^NS,a^
Abikoff et al. 2007 [1]	4 weeks^b^	3–5.5	MPH (1.25, 2.5, 5.0 or 7.5 mg t.i.d., optimized)	114 (1:1)	SNAP-IV parent–teacher composite score (effect size 1.20***) [49]	SCS-T total score (effect size 0.39*)	SCS-P total score (effect size 0.13^NS^) SSRS-P Total Social Skills (effect size 0.14^NS^)
Short-term studies—stimulant and non-stimulant medications							
Newcorn et al. 2008 [68]	6 weeks	6–16	ATX (0.8–1.8 mg/kg/day) or OROS-MPH (18–54 mg/day)	516 (3:3:1)	ADHD-RS-IV total score (effect sizes ATX, 0.6**; OROS-MPH, 0.8***)	CHQ^a,c^ Psychosocial summary (ATX,*^,d^ OROS-MPH*^,d^)	
Short-term studies—non-stimulant medications							
Hervas et al. 2014 [51]	10 weeks (children) or 13 weeks (adolescents)	6–17	GXR (1–7 mg/day, optimized); ATX reference (0.5–1.4 mg/kg/day)	338 (1:1:1)	ADHD-RS-IV total score (effect sizes GXR, 0.76***; ATX, 0.32*)	WFIRS-P total score (effect sizes GXR, 0.44**; ATX, 0.28*), Learning and School (0.42**; 0.32*), Family (GXR, 0.38**), Social Activities (GXR, 0.45**)	WFIRS-P Family (effect size ATX, 0.16^NS^), Social Activities (ATX, 0.21^NS^), Life Skills (GXR, 0.23^NS^; ATX, 0.16^NS^), Child’s Self-Concept (0.09^NS^; 0.15^NS^), Risky Activities (0.21^NS^; 0.14^NS^)
Wilens et al. 2013 [100]^e^	9 weeks	6–17	Adjunctive GXR (a.m. or p.m., 1–4 mg/day, optimized) to current long-acting stimulant	461 (1:1:1)	ADHD-RS-IV total score (effect sizes a.m., 0.377**; p.m., 0.447***) [98]	Parent-rated BSFQ^a^ score (a.m.*** and p.m.** dosing)	Participant-rated BSFQ^a^ Feeling^NS^, Behaviour^NS^
Wilens et al. 2015 [101]	13 weeks	13–17	GXR (1–7 mg/day, optimized)	314 (1:1)	ADHD-RS-IV total score (effect size 0.52***)	None	WFIRS-P Learning and School (effect size 0.22^NS^), Family (0.11^NS^)
Stein et al. 2015 [86]	8 weeks	6–12	GXR (a.m. or p.m., 1–4 mg/day, optimized)	333 (1:1:1)	ADHD-RS-IV total score (overall effect size 0.77***) [70]	WFIRS-P total score (overall effect size 0.448***), Family (0.528***), Learning and School (0.463***), Academic Performance (0.413**), Social Activities (0.419***), Risky Activities (0.337*)	WFIRS-P Behaviour in School (overall effect size 0.389^NS^), Life Skills (0.166^NS^), Child’s Self-Concept (0.052^NS^)
Wehmeier et al. 2011 [94]^f^	9 weeks	6–17	ATX (1.2 mg/kg/day, fast or slow forced titration)^g^	181 (1:1:1)	SNAP-IV ADHD subscale score (overall effect size 0.72***) [38]	KINDL-R total score (overall effect size 0.377*), Physical Well-Being (−0.390*), Emotional Well-Being (0.318*), Self-Esteem (0.590***), Family (0.395*), Friends (0.387*)	KINDL-R School (overall effect size 0.248^NS^)
Svanborg et al. 2009 [91]	10 weeks	7–15	ATX (1.2 mg/kg/day, forced titration)^h^	99 (1:1)	Parent-rated ADHD-RS-IV total score*** [90]	CHIP-CE:PRF^a^ Achievement*, Risk Avoidance*	CHIP-CE:PRF^a^ Resilience^NS^, Satisfaction^NS^ and Comfort^NS^ JTJA total scores^NS,i^ and domains^NS^
Dell’Agnello et al. 2009 [37]^f^	8 weeks	6–15	ATX (1.2 mg/kg/day, forced titration)	139 (3:1)	SNAP-IV ADHD subscale score***	CHIP-CE:PRF^a^ Risk Avoidance*	CHIP-CE:PRF^a^ Achievement^NS^, Satisfaction^NS^, Resilience^NS^, Comfort^NS^
Escobar et al. 2009 [42]	12 weeks	6–15	ATX (1.2 mg/kg/day, forced titration)	151 (2:1)	Parent-reported, investigator-rated ADHD-RS-IV total score (effect size 0.82***) [67]	CHIP-CE:PRF^a^ Risk Avoidance (effect size 0.557***), Achievement (0.286*) CHIP-CE:SRF/AE^a^ Risk Avoidance (effect size 0.387**)	CHIP-CE:PRF^a^ Satisfaction (effect size 0.033^NS^), Comfort (0.163^NS^), Resilience (0.113^NS^) CHIP-CE:SRF/AE^a^ (self-reported) Satisfaction (effect size 0.137^NS^), Comfort (0.104^NS^), Resilience (0.016^NS^), Achievement (0.085^NS^)
Brown et al. 2006 [17]	7 weeks	8–12	ATX (0.8–1.8 mg/kg/day, optimized)	153 (2:1)	ADHD-RS-IV teacher-rated version total score (effect size 0.62**)	Frequency of CHQ-PF50^a^ Psychosocial summary response*	CHQ-PF50^a^ Psychosocial summary (effect size 0.32^NS^) APRS total score (effect size 0.31^NS^)
Michelson et al. 2001 [66]	8 weeks	8–18	ATX (0.5, 1.2 or 1.8 mg/kg/day, forced titration)	297 (1:2:2:2)	Parent-rated ADHD-RS-IV total score (0.5 mg/kg^NS^; 1.2 mg/kg***; 1.8 mg/kg***)	CHQ-PF50^a,c^ Psychosocial summary* (all doses)	CHQ-PF50^a^ Physical summary^NS^ (all doses)
Long-term studies—stimulant medications							
Banaschewski et al*. 2*014 [14]	6 weeks (withdrawal after 6 months)	6–17	LDX (30, 50 or 70 mg/day, optimized)	153 (1:1)	Treatment failure (≥50% increase in ADHD-RS-IV total score and ≥2-point increase in CGI-S score)***; ADHD-RS-IV total score (effect size 1.493***)	CHIP-CE:PRF^a^ Achievement (effect size 0.696***), Risk Avoidance (0.829***), Satisfaction (0.636***) WFIRS-P total score (0.908***), Family (0.859***), Learning and School (0.716***), Risky Activities (0.506**)	CHIP-CE:PRF^a^ Resilience (effect size 0.275^NS^), Comfort (0.348^NS^) WFIRS-P Life Skills (0.228^NS^), Child’s Self-Concept (0.203^NS^), Risky Activities (0.197^NS^)
Long-term studies—non-stimulant medications							
Michelson et al. 2004 [65]	6 months (withdrawal)	6–15	ATX (1.2–1.8 mg/kg/day, optimized)	416 (2:1)	Relapse avoidance (ADHD-RS-IV total score ≥ 90% of baseline value and ≥ 2-point increase in CGI-S score)**; ADHD-RS-IV total score***	CHQ^a,c^ Psychosocial summary*	None

Positive effect sizes indicate positive effects of medication on HRQoL/functioning versus placebo

*ADHD* attention-deficit/hyperactivity disorder, *ADHD*-*RS*-*IV* ADHD Rating Scale IV (investigator-rated unless otherwise stated), *APRS* Academic Performance Rating Scale, *ATX* atomoxetine, *BSFQ* Before-School Functioning Questionnaire, *CADS*-*T* Conners’ ADHD/DSM-IV Scale-Teacher version, *CGI*-*S* Clinical Global Impressions-Severity, *CHIP*-*CE:PRF* Child Health and Illness Profile-Child Edition: Parent Report Form, *CHIP*-*CE:SRF/AE* Child Health and Illness Profile-Child Edition: Self-Report Form/Adolescent Edition, *CHQ* Child Health Questionnaire, *CHQ*-*PF50* Child Health Questionnaire-50-item Parent Form, d-MPH-ER dexmethylphenidate extended release, *GXR* guanfacine extended release, *HRQoL* health-related quality of life, *JTJA* Jag Tycker Jag Är [I think I am], *KINDL*-*R* Revidierter Fragebogen für Kinder und Jugendliche zur Erfassung der gesundheitsbezogenen Lebensqualität [Revised questionnaire to assess health-related quality of life in children and adolescents], *LDX* lisdexamfetamine, *MPH* methylphenidate, *NS* not significant, *NT* not tested, *OROS*-*MPH* osmotic-release oral system methylphenidate, *SCS*-*P/T* Social Competence Scale-Parent/Teacher, *SNAP*-*IV* Swanson, Nolan, and Pelham Rating Scale-Revised, *SSRS*-*P/T* Social Skills Rating System-Parent/Teacher, *TD*-*MPH* transdermal methylphenidate, *t.i.d.* three times daily, *WFIRS*-*P* Weiss Functional Impairment Rating Scale-Parent, *YQOL*-*R* Youth Quality of Life Instrument-Research Version

* *p* < 0.05, ** *p* < 0.01, *** *p* < 0.001 (active treatment vs placebo)

^a^CHIP-CE:PRF subdomains, CHQ-PF50 concepts and BSFQ individual items are not included in this summary

^b^Following 5-week titration (randomized, double-blind, within-subject, crossover period)

^c^Assumed to be CHQ-PF50 and not the 28-item version

^d^The publication states that the differences were significant, but do not supply the *p* values

^e^Patients with suboptimal response to previous treatment with an extended release psychostimulant

^f^Patients with comorbid oppositional defiant disorder

^g^Two ATX groups pooled for analysis (shown here) and also analysed separately (not shown here)

^h^All patients’ parents received psychoeducation

^i^Total scores on the JTJA Low scale (children aged 7–10 years) and Medium–High scale (children aged 11–16 years)

**Table 2:** Overview of HRQoL and functional outcomes from the included randomized placebo-controlled studies of medications in adults with ADHD

References	Duration of randomized assessment period	Age, years	Active treatment group(s)	Number randomized (ratio, active[s]: placebo)	Principal symptom-based efficacy outcome versus placebo at study endpoint or last assessment	HRQoL/functional outcomes: significant effect(s) of medication(s) versus placebo at study endpoint or last assessment	HRQoL/functional outcomes: other reported comparisons of medication(s) versus placebo
Short-term studies—stimulant mediations							
Huss et al. 2014 [52]	9 weeks^a^	18 − 60	MPH-LA (40, 60 or 80 mg/day, fixed)	725 (1:1:1:1)	ADHD-RS-IV total score (effect size 0.55***)	SDS total score (effect size 0.39*)	None (SDS subscales not reported)
Adler et al. 2013 [3]^b^	10 weeks	18 − 55	LDX (30, 50 or 70 mg/day, optimized)	161 (1:1)	ADHD-RS-IV total score (effect size 0.94***) [4]	AIM-A Performance and Daily Functioning (effect size 0.93*), Impact: Daily Interference (0.62*), Impact: Bother/Concern (0.57*), Relationships/Communication (0.31*), Living with ADHD (0.79***), General Well-Being (0.70***), Overall QoL questions 1 (0.29*) and 4 (0.44***)	AIM-A overall QoL questions 2^NT^ and 3^NT^ AAQoL total score, Life Productivity^NT^, Psychological Health^NT^, Life Outlook^NT^, Relationships^NT^
Casas et al. 2013 [25]	12 weeks	18–65	OROS-MPH (54 or 72 mg/day, fixed)	279 (1:1:1)	CAARS-O:SV total score (effect sizes 54 mg, 0.20^NS^; 72 mg 0.49**)	AIM-A Performance and Daily Functioning (54 mg**, 72 mg***), Impact: Daily Interference (54 mg*, 72 mg*), Relationships/Communication (72 mg**), Living with ADHD (72 mg*), General Well-Being (54 mg*)	AIM-A Relationships/Communication (54 mg^NS^), Living with ADHD (54 mg^NS^), General Well-Being (72 mg^NS^), Impact: Bother/Concern (54 mg^NS^, 72 mg^NS^); overall QoL questions not reported SDS total^NS^ and subscale^NS^ scores
Rösler et al. 2013 [82]	5 weeks	18–65	OROS-MPH (18, 36 or 72 mg/day, fixed)	402 (1:1:1:1)	CAARS-O:SV total score (18 mg*, 36 mg*, 72 mg***) [64]	SDS total score (18 mg**, 36 mg*, 54 mg**), Work (18 mg**, 54 mg**), Social Life (18 mg*, 36 mg**, 54 mg***), Family Life (18 mg*, 54 mg**)	SDS Work (36 mg^NS^), Family Life (36 mg^NS^) Q-LES-Q^NS^
Weiss et al. 2012 [96]	20 weeks	18–66	*d*-AMP (5–20 mg b.i.d; both groups also received CBT)	48 (1:1)^c^	ADHD-RS-IV total score^NS^	None	SDS^NS^ (subscales not reported)
Retz et al. 2012 [78]	8 weeks	≥18	MPH-ER (40, 60, 80 or 120 mg b.i.d., optimized)	162 (1:1)	WRAADDS total score (effect size 0.54***)	SDS total score (effect size 0.40*)	None (SDS subscales not reported)
Spencer et al. 2008a and b [84, 85]	7 weeks	18–55	MAS-XR3 (12.5, 25, 50 or 75 mg/day, optimized)	274 (1:1)	ADHD-RS-IV total score***	AIM-A Living with ADHD***, General Well-Being***, Performance and Daily Functioning***, Relationships/Communication***, Impact: Bother/Concern*, Impact: Daily Interference**, overall QoL questions 1*** and 4***	AIM-A overall QoL questions 2^NT^ and 3^NT^
Short-term studies—non-stimulant medications							
Goto et al. 2013 [48]	10 weeks	≥18	ATX (40, 80, 105 or 120 mg/day, optimized)	391 (1:1)	CAARS-I:SV total score (effect size 0.55***)	AAQoL total score**, Life Outlook*, Life Productivity*** and Relationships**	AAQoL Psychological Health^NS^
Durell et al. 2013 [40]	12 weeks	18–30	ATX (40, 80 or 100 mg b.i.d., optimized)	445 (1:1)	CAARS-I:SV total score (effect size 0.4***)	AAQoL total score (effect size 0.3**), Relationships*, Life Productivity**, Psychological Health*	AAQoL Life Outlook^NS^ SASS total score^NS^
Lee et al. 2014 [55]	10 weeks	≥18	ATX (40–120 mg/day)	74 (1:1)	CAARS-I:SV total score***	None	AAQoL total score^NS^, Psychological Health^NS^, Life Outlook^NS^, Life Productivity^NS^, Quality of Relationships^NS^
Adler et al. 2009a [5]^d^	14 weeks	18–65	ATX (40, 80 or 100 mg/day, optimized)	442 (1:1)	CAARS-I:SV total score (effect size 0.47***)	AAQoL total score (effect size 0.24*), Psychological Health*	AAQoL Life Outlook^NS^, Life Productivity^NS^, Quality of Relationships^NS^
Manor et al. 2012 [59]	6 weeks	18–50	Metadoxine (1.4 g/day)	120 (1:1)	CAARS-I total score (effect size 0.4*)	AAQoL total score*	None (AAQoL domains not reported)
Riahi et al. 2010 [79]	6 weeks	Adults	Reboxetine (4 mg b.i.d.)	46 (1:1)	CAARS-S:SV total score (effect size 0.04^NS^)	GAF score***	None
Long-term studies—non-stimulant medications							
Wietecha et al. 2012 [97]^e^	24 weeks	≥18	ATX (60, 80 or 100 mg/day, optimized)	502 (1:1)	CAARS-I:SV total score (effect size 0.57***)	DAS Affectional Expression* PSI Parent Domain Depression*, Life Stress*	DAS Total Dyadic Adjustment^NS^, Dyadic Consensus^NS^, Dyadic Satisfaction^NS^, Dyadic Cohesion^NS^ PSI Total Stress^NS^, Child Domain Total^NS^, Parent Domain Total^NS^ APQ (Patient)^NS^ (all domains) PSCS^NS^ (all domains) FAM-III^NS^ (all domains)
Adler et al. 2009b [6]	6 months	18–54	ATX (25, 40, 80 or 100 mg/day, optimized)	501 (1:1)	AISRS total score**	AAQoL total score**, Life Productivity**, Psychological Health*, Quality of Relationships*	AAQoL Life Outlook^NS^
Adler et al. 2008 [7]	6 months	18–50	ATX (40, 80 or 100 mg/day, flexible)	410 (2:1)	CAARS-S:SV total score*^,f^	AAQoL Life Outlook* DBS (observer reported subset, *n* = 252)*	AAQoL total score^NS^, Life Productivity^NS^, Relationships^NS^, Psychological Health^NS^ EWPS total score^NS,f^ DBS (self-reported)^NS^

Positive effect sizes indicate positive effects of medication on (HR)QoL/functioning versus placebo

*AAQoL* Adult ADHD Quality of Life, *ADHD* attention-deficit/hyperactivity disorder, *ADHD*-*RS*-*IV* ADHD Rating Scale IV (investigator-rated unless otherwise stated), *AIM*-*A* ADHD Impact Module-Adult, *AISRS* Adult ADHD Investigator Symptom Rating Scale, *APQ* Alabama Parenting Questionnaire, *ATX* atomoxetine, *b.i.d.* twice daily, *CBT* cognitive behavioural therapy, *CAARS*-*I:SV* Conners’ Adult ADHD Rating Scale-Investigator: Screening Version, *CAARS*-*O:SV* Conners’ Adult ADHD Rating Scale-Observer: Screening Version, *CAARS*-*S:SV* Conners’ Adult ADHD Rating Scale- Self-rated: Screening Version, d-AMP dextroamphetamine, *DAS* Dyadic Adjustment Scale, *DBS* Driving Behavior Survey, *EWPS* Endicott Work Productivity Scale, *FAM*-*III* Family Assessment Measure III Dyadic Relationship Scales, *GAF* Global Assessment of Functioning, *HRQoL* health-related quality of life, *LDX* lisdexamfetamine, *MAS*-*XR3* triple-bead mixed amphetamine salts extended release, *MPH*-*ER* methylphenidate extended release, *MPH*-*LA* long-acting methylphenidate, *NS* not significant, *NT* not tested, *OROS*-*MPH* osmotic-release oral system methylphenidate, *PSCS* Parenting Sense of Competence Scale, *PSI* Parenting Stress Index, *Q*-*LES*-*Q* Quality of Life Enjoyment and Satisfaction Questionnaire, *QoL* quality of life, *SASS* Social Adaptation Self-evaluation Scale, *SDS* Sheehan Disability Scale, *WRAADDS* Wender–Reimherr Adult Attention Deficit Disorder Scale

* *p* < 0.05, ** *p* < 0.01, *** *p* < 0.001 (active treatment vs placebo)

^a^No HRQoL/functional outcomes reported in the 9-month period

^b^Patients with ADHD and executive function deficits. Primary efficacy outcome measure: Behaviour Rating Inventory of Executive Function-Adult (ADHD-RS-IV secondary)

^c^A third group of patients who received antidepressant medication plus CBT was not included in this pre-specified analysis

^d^Patients with comorbid social anxiety disorder

^e^Patients in a reciprocal heterosexual relationship and living with at least one child aged 6–17 years

^f^EWPS was the primary efficacy outcome and showed a significant effect of ATX versus placebo at 1 month, but not at endpoint. Of four symptom-based outcome measures, only the self-rated instrument showed a significant effect of ATX versus placebo

### Patients, medications and outcomes assessed

Of the 34 included studies, 18 were conducted in children aged 3–12 years and/or adolescents aged 13–18 years (Table 1), and 16 were conducted in adults aged 18 years or older (Table 2). None of the studies selected patients based on measures of HRQoL or functional impairment at baseline, but nearly all recruited patients with at least moderately severe ADHD symptoms (e.g. ADHD-RS-IV total score ≥28). Fourteen studies investigated stimulant medications (amphetamines, including LDX, and various formulations of MPH), and 21 studies investigated non-stimulant medications (ATX, guanfacine extended release [GXR], metadoxine and reboxetine). Three studies investigated more than one medication. With the exception of metadoxine and reboxetine, all of the medications investigated in the studies are approved for treatment of ADHD in one or more countries.

Twenty-nine studies were short-term double-blind studies (≤20 weeks); two were long-term (≥6 months) studies in children and adolescents (both double-blind, randomized withdrawal studies); and three were long-term double-blind studies in adults (Tables 1, 2). Of the 18 studies in children and adolescents, 12 assessed HRQoL and 9 assessed functional impairment; of the 16 adult studies, 12 assessed HRQoL and 11 assessed functional impairment (Table 3).

**Table 3:** Instruments used to measure HRQoL/functional outcomes

	Generic (rater)	ADHD-specific (rater)	
Studies in children and adolescents			
HRQoL	CHIP-CE:PRF (parent) – 5 studies [14, 15, 37, 42, 91] CHQ-PF50 (parent)^a^—5 studies [17, 50, 65, 66, 68] YQOL-R (patient)—1 study [45] KINDL-R (parent)—1 study [94] JTJA (patient)—1 study [91] CHIP-CE:SRF/AE (patient)—1 study [42]		
Functional impairment	SCS-P/T (parent/teacher); SSRS-P/T (parent/teacher)—1 study [1] APRS (teacher)—1 study [17]	WFIRS-P (parent)—5 studies [14, 15, 51, 86, 101] BSFQ (patient/parent; investigator)—2 studies [99, 100]	
Studies in adults			
HRQoL	Q-LES-Q (patient)—1 study [82]	AAQoL (patient)—8 studies [3, 5–7, 40, 48, 55, 59]	
Functional impairment with HRQoL element		AIM-A (patient)—3 studies [3, 25, 84, 85]	
Functional impairment	SDS (patient)—5 studies [25, 52, 78, 82, 96] GAF (investigator)—1 study [79] DBS (patient or observer)—1 study [7] EWPS (patient)—1 study [7] Various family functioning measures (patient)^b^—1 study [97]		

^a^Parent version of CHQ assumed (not specified in article) [68]

^b^DAS (patient), PSI (patient), PSCS (patient), FAM-III (patient) and APQ-Patient (patient)

*AAQoL* Adult ADHD Quality of Life, *ADHD* attention-deficit/hyperactivity disorder, *AIM*-*A* ADHD Impact Module-Adult, *APQ* Alabama Parenting Questionnaire, *APRS* Academic Performance Rating Scale, *BSFQ* Before-School Functioning Questionnaire, *CHIP*-*CE:PRF* Child Health and Illness Profile-Child Edition: Parent Report Form, *CHIP*-*CE:SRF/AE* Child Health and Illness Profile-Child Edition: Self-Report Form/Adolescent Edition, *CHQ*-*PF50* Child Health Questionnaire-50-item Parent Form, *DAS* Dyadic Adjustment Scale, *DBS* Driving Behavior Survey, *EWPS* Endicott Work Productivity Scale, *FAM*-*III* Family Assessment Measure III Dyadic Relationship Scales, *GAF* Global Assessment of Functioning, *HRQoL* health-related quality of life, *JTJA* Jag Tycker Jag Är [I think I am], *KINDL*-*R* Revidierter Fragebogen für Kinder und Jugendliche zur Erfassung der gesundheitsbezogenen Lebensqualität [Revised questionnaire to assess health-related quality of life in children and adolescents], *Q*-*LES*-*Q* Quality of Life Enjoyment and Satisfaction Questionnaire, *PSCS* Parenting Sense of Competence Scale, *PSI* Parenting Stress Index, *SCS*-*P/T* Social Competence Scale-Parent/Teacher, *SDS* Sheehan Disability Scale, *SSRS*-*P/T* Social Skills Rating System-Parent/Teacher, *WFIRS*-*P* Weiss Functional Impairment Rating Scale-Parent, *YQOL*-*R* Youth Quality of Life Instrument-Research Version

HRQoL in children and adolescents was always assessed using generic instruments, but HRQoL in adults was mostly assessed using ADHD-specific instruments. Conversely, functional impairment was mostly assessed using ADHD-specific instruments in children and adolescents, but generic instruments in adults. HRQoL in adults was self-rated in all studies, but proxy-rated (by parents) in all but three studies in children and adolescents (two in adolescents [42, 45] and one in children/adolescents [91]) (Table 3).

### Baseline impairment

Some of the studies in children and adolescents used generic HRQoL instruments that have been standardized to community norms or for which reference population data are available. These can provide an indication of the burden of untreated ADHD when patients are assessed at baseline. The domains with the greatest deficits may reflect impairments characteristic of the disorder and may also be considered as potential targets for treatment.

**Fig. 3:**
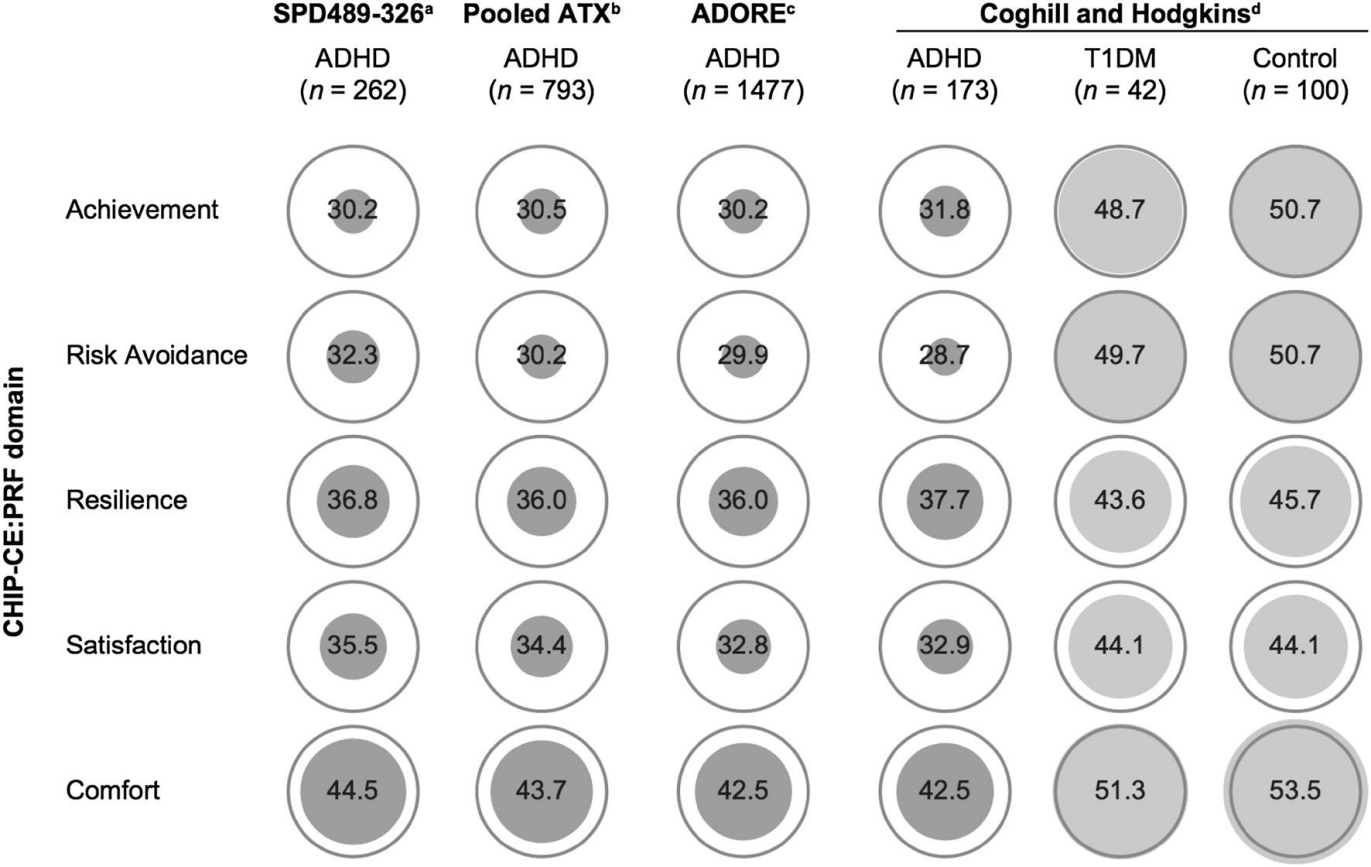
Pre-treatment baseline CHIP-CE:PRF T-scores in children and adolescents with ADHD. T-scores have a mean of 50 and a standard deviation of 10. Circle diameter is proportional to T-score, with a diameter of zero corresponding to a T-score of 20. Rings indicate the mean in the reference population. ^a^Study SPD489-326 [15] (included in this review) involved mainly the same patients as the short-term LDX study SPD489-325 [14] (also included in this review), so only the former is shown. ^b^Pooled analysis of five ATX studies [43]: three randomized placebo-controlled trials (included in this review) [37, 42, 91], and two open-label studies (not included in this review). ^c^Observational study (not included in this review), shown for comparison [80]. ^d^Observational study with non-ADHD control groups (not included in this review), shown for comparison [32]. ADHD attention-deficit/hyperactivity disorder, *ADORE* Attention-Deficit/Hyperactivity Disorder Observational Research in Europe, *ATX* atomoxetine, *CHIP*-*CE:PRF* Child Health and Illness Profile-Child Edition: Parent Report Form, *LDX* lisdexamfetamine, *T1DM* type 1 diabetes mellitus

The Child Health and Illness Profile-Child Edition: Parent Report Form (CHIP-CE:PRF) is a generic, parent-rated measure of children’s HRQoL in five domains (Achievement, Risk Avoidance, Resilience, Satisfaction and Comfort). Baseline CHIP-CE:PRF T-scores from five included studies (two of LDX and three of ATX) were strikingly consistent in indicating that children and adolescents with ADHD have substantially impoverished HRQoL before treatment, especially in the Achievement and Risk Avoidance domains [14, 15, 37, 42, 91]. Impairment was less marked in the domains of Resilience and Satisfaction, and scores in Comfort were close to the community norm [14, 15, 37, 42, 91]. These findings are also consistent with the results of two observational population studies (not included in this review) that assessed the impact of ADHD on HRQoL using the CHIP-CE:PRF (Fig. 3) [32, 80].

The German, parent-rated *Revidierter Fragebogen für Kinder und Jugendliche zur Erfassung der gesundheitsbezogenen Lebensqualität* (KINDL-R; revised questionnaire to assess health-related quality of life in children and adolescents) also assesses children’s HRQoL overall and in six domains, namely Physical Well-Being, Emotional Well-Being, Self-Esteem, Friends, Family and School. In one included study [94], overall mean pre-treatment KINDL-R scores in children and adolescents with ADHD were substantially lower than the mean in the German reference population [22] [62.9 (95% confidence interval: 61.0–64.8) vs 78.2 (77.8–78.7) on a scale of 0–100], with the largest deficits in the domains of Family and Friends, and no deficit in the Physical Well-Being domain. The parent-rated Child Health Questionnaire-50-item Parent Form (CHQ-PF50) provides Psychosocial and Physical summary scores. In one included study, the mean baseline Psychosocial T-score in children and adolescents with ADHD was almost two standard deviations below the normative mean (31.3–35.4 vs 50) [66].

Considered together, baseline parent-rated HRQoL data from the included child/adolescent studies indicate that parent-rated HRQoL of children with ADHD is approximately 1.5–2.0 standard deviations lower than that of control populations in domains reflecting achievement and risk-taking, as was also found in a previous systematic review [34]. HRQoL deficits may be less marked with self-rated than with parent-rated instruments in children and adolescents. In one included study, mean T-scores on the self-rated Child Health and Illness Profile-Child Edition: Self-Report Form or Adolescent Edition (CHIP-CE:SRF/AE) was not as far below the normative mean as those on the CHIP-CE:PRF [42]. This agrees with an observational study (not included in this review) in which self-rated CHIP-CE:SRF scores correlated only moderately with parent-rated CHIP-CE:PRF T-scores [32]. In another included study [45], mean scores on the self-rated Youth Quality of Life Instrument-Research Version (YQOL-R) were not far below the mean for community norms [73] (79.2–79.5 vs 82.2 on a 0–100 scale).

The other multi-domain instruments used in the studies included in this review do not have reference population data. However, some domains consistently had worse pre-treatment scores than others. Weiss Functional Impairment Rating Scale-Parent (WFIRS-P) baseline scores were generally worst in the Learning and School domain and in the Family domain in children and adolescents. In adults, ADHD Impact Module-Adult (AIM-A) scores were generally worst in the Performance and Daily Functioning domain, and Adult ADHD Quality of Life (AAQoL) scores were generally worst in the Productivity domain.

### Effectiveness of pharmacological treatment in children and adolescents

#### ADHD symptoms in children and adolescents

All of the 18 studies in children and/or adolescents demonstrated significant beneficial effects of medication on ADHD symptoms compared with placebo (Table 1). Effect sizes of active treatment versus placebo were reported for 12 studies and were calculated using published data from four studies. Effect sizes of at least 0.5 (an approximate indicator of minimum clinically important difference) [71] for ADHD symptom outcomes in at least one active treatment group were observed in all of these 16 studies except the long-term ATX withdrawal study [65] and the adjunctive GXR study [100]. Effect sizes for symptom-based outcomes were generally lower for non-stimulants (range 0.32–1.20) than for stimulants (range 0.80–1.80) (Table 4).

**Table 4:**
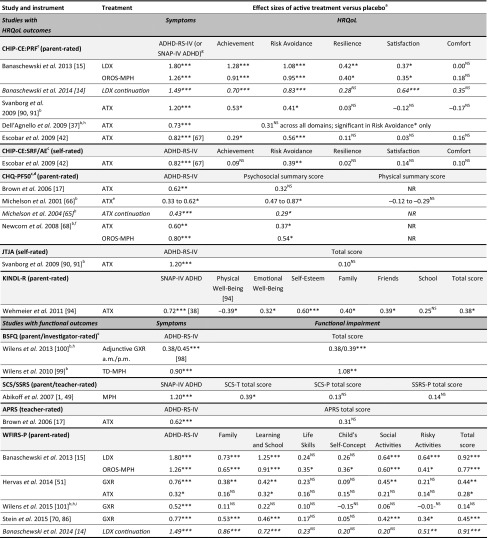
Summary of treatment effect sizes in children and adolescents

Effect sizes have been rounded to 2 decimal places. Positive effect sizes indicate a beneficial effect of treatment compared with placebo. Italics indicate long-term randomized withdrawal studies

*ADHD* attention-deficit/hyperactivity disorder*, ADHD*-*RS*-*IV* ADHD Rating Scale IV (investigator-rated unless otherwise stated), *APRS* Academic Performance Rating Scale, *ATX* atomoxetine, *BSFQ* Before-School Functioning Questionnaire, *CHIP*-*CE:SRF/AE* Child Health and Illness Profile-Child Edition: Self-Report Form/Adolescent Edition, *CHIP*-*CE:PRF*, Child Health and Illness Profile-Child Edition: Parent Report Form, *CHQ*-*PF50* Child Health Questionnaire-50-item Parent Form, *GXR* guanfacine extended release, *HRQoL* health-related quality of life, *JTJA* Jag Tycker Jag Är [I think I am], *KINDL*-*R* Revidierter Fragebogen für Kinder und Jugendliche zur Erfassung der gesundheitsbezogenen Lebensqualität [Revised questionnaire to assess health-related quality of life in children and adolescents], *LDX* lisdexamfetamine, *MPH* methylphenidate, *NR* not reported, *NS* not significant, *OROS*-*MPH* osmotic-release oral system methylphenidate, *SCS*-*P/T* Social Competence Scale-Parent/Teacher, *SD* standard deviation, *SNAP*-*IV* Swanson, Nolan, and Pelham Rating Scale-Revised, *SSRS*-*P* Social Skills Rating System-Parent, *TD*-*MPH* transdermal methylphenidate, *WFIRS*-*P* Weiss Functional Impairment Rating Scale-Parent

* *p* < 0.05; ** *p* < 0.01; *** *p* < 0.001 (active treatment vs placebo)

^a^Effect sizes could not be calculated for Findling et al. 2011 [45], the only study to use the Youth Quality of Life Instrument-Research Version

^b^Effect sizes of active medication versus placebo were calculated using published data (mean change, n, and SD or standard error of the mean; or n and F-statistic [as appropriate])

^c^CHQ-PF50 concepts, CHIP-CE:PRF subdomains and BSFQ individual items were not included

^d^Effect sizes could not be calculated for Greenhill et al. 2006 [50]

^e^ATX doses of 0.5, 1.2 or 1.8 mg/kg/day; results shown as a range across the three doses

^f^Publication does not specify which version of the CHQ was used; parent version is assumed

^g^Symptom measure was ADHD-RS-IV for all CHIP-CE:PRF studies except Dell’Agnello et al. 2009 [37], which used SNAP-IV ADHD

^h^Effect sizes were calculated from published data for the symptomatic measure (ADHD-RS-IV or SNAP-IV ADHD) but from the corresponding ClinicalTrials.gov entry for the functional/HRQoL measure (CHIP-CE:PRF or BSFQ); CHIP-CE:PRF domain effect sizes could not be calculated for Dell’Agnello et al. 2009 [37]

^i^Effect sizes were published for the WFIRS-P Learning and School domain and Family domain and were calculated from published data for the remaining domains

#### HRQoL in children and adolescents

Of the 12 studies that assessed HRQoL, 10 reported significant placebo-adjusted effects of medication in at least one domain or cross-domain summary statistic, all using patient proxy ratings (Table 1). Effect sizes of active treatment versus placebo were available for all but one of these studies [50] and were 0.5 or above in at least one domain in seven of the remaining nine [14, 15, 42, 66, 68, 91, 94].

Seven studies used multi-domain instruments (e.g. CHIP-CE:PRF, KINDL-R, CHQ-PF50) to assess changes in HRQoL with medication. Effect sizes varied across domains and were generally larger in domains relating to risk-taking and achievement than in domains relating to physical components of HRQoL in patients treated with ADHD medications (Table 4). In the most responsive domains, effect sizes observed for HRQoL were generally larger for stimulants (range 0.54–1.28) than for non-stimulants (0.29–0.87).

The CHIP-CE:PRF was used in five studies: three short-term studies of ATX [37, 42, 91], a short-term study of LDX with an osmotic-release oral system MPH (OROS-MPH) reference arm [15], and a long-term, randomized withdrawal study of LDX (Table 4) [14]. In all of the short-term studies, the largest effect sizes for active treatment versus placebo were in the Risk Avoidance and Achievement domains. The improvements in these domains with short-term LDX treatment [15] were maintained with continued treatment in the subsequent randomized withdrawal study [14]. Results in the Resilience and Satisfaction domains differed across studies, with significant effects and small or moderate effect sizes in the LDX studies but not in the ATX studies. In the Comfort domain, no significant effects of treatment were reported in any study [14, 15, 37, 42, 91]. In the study of ATX using the KINDL-R, significant beneficial effects of ATX were reported in all domains except School and Physical Well-Being, with a significant effect in favour of placebo in the latter [94]. This suggests that the KINDL-R Physical Well-Being domain may be more sensitive to side effects of medication than other instruments [94].

Three of the five studies that used the CHQ-PF50 reported significant placebo-adjusted improvements in Psychosocial summary score, with effect sizes of 0.29–0.87 for ATX and 0.54 for OROS-MPH (the reference arm in one ATX study, in which the ATX effect size was 0.37) (Table 4) [65, 66, 68]. Effect sizes were not available for one [50]. In the remaining study, the improvement in Psychosocial summary score with ATX treatment was not significant, but a significantly greater proportion of patients receiving ATX were classified as CHQ-PF50 responders, compared with placebo (43.8 vs 22.2%; *p* < 0.04) [17]. The effect size of 0.32 and relatively small number of patients (*N* = 153) suggests that this study may have been statistically underpowered. Physical summary score was reported in only one of the studies to use the CHQ-PF50 [66], which showed negative and non-significant effect sizes of ATX versus placebo (Table 4).

#### Functional impairment in children and adolescents

Five of the nine studies that assessed functional impairment in children and adolescents used the parent-rated WFIRS-P. Four of these five studies (three short-term studies and one randomized withdrawal study) reported significant placebo-adjusted effects of treatment on WFIRS-P total score (Table 4) [14, 15, 51, 86]. Scores in the Family domain and the Learning and School domain were most consistently improved relative to placebo in these studies. Effects were not consistent in other domains, although scores in the Life Skills and Child’s Self-Concept domains were the least responsive to treatment (Table 4). In the most responsive domains, WFIRS-P effect sizes were larger for stimulants (range 0.86–1.25) than for non-stimulants (0.32–0.58). Three studies reported significant effects of medications on single-domain functional impairment measures (Table 1) [1, 99, 100], with an effect size above 0.5 in a study of transdermal MPH (TD-MPH) using the Before-School Functioning Questionnaire (BSFQ) [99].

#### Patient self-rating and proxy ratings in children and adolescents

Results from several studies suggest that observed treatment effects may be larger when questionnaires are completed by proxies than by patients, both for instruments described as HRQoL measures and those described as functional impairment measures. This mirrors the greater baseline HRQoL deficits observed with parent-rated than with self-rated HRQoL instruments.

The self-rated CHIP-CE:SRF/AE and parent-rated CHIP-CE:PRF were used together in one study of ATX [42]. The pattern of changes observed across the five domains was similar with the CHIP-CE:SRF/AE and the CHIP-CE:PRF, but effect sizes were smaller with the self-rated than with the parent-rated instrument in all domains except Satisfaction (Table 4). An effect size above 0.5 for ATX versus placebo was seen only in the CHIP-CE:PRF Risk Avoidance domain in this study. In another of the ATX studies that used the CHIP-CE:PRF, the significant effects of ATX versus placebo in the Achievement domain and the Risk Avoidance domain (effect sizes, 0.53 and 0.41, respectively; Table 4) were not reflected in a one-dimensional self-rated Swedish HRQoL scale (effect size 0.10). It is possible that this scale, the *Jag Tycker Jag Är* [I think I am] (JTJA), may not be sensitive to HRQoL deficits in patients with ADHD [91]. Similarly, self-rated YQOL-R scores did not significantly improve from baseline to endpoint in either the LDX or placebo groups (and did not differ between groups) of a study in adolescents, despite an effect size for LDX 70 mg of 0.72 (*p* ≤ 0.0056) on ADHD symptoms in the same study [45].

Two studies used the BSFQ, an ADHD-specific instrument with a proxy-rated section (completed by investigators or parents; Table 4) and a self-rated section (completed by patients in collaboration with their parents). In one study, significantly greater improvement with TD-MPH than with placebo was observed using the investigator-rated BSFQ, with an effect size of 1.08 (*p* < 0.01), but not using the self-reported BSFQ [99]. Similarly, significantly greater improvement with GXR than with placebo as an adjunct to stimulant medication was observed using the parent-rated BSFQ (effect size 0.38–0.39; *p* < 0.001), but not using the self-rated BSFQ [100].

### Effectiveness of pharmacological treatment on HRQoL and functional impairment in adults

#### ADHD symptoms in adults

Of the 16 included studies conducted in adults, 14 demonstrated significant beneficial effects of medication on ADHD symptoms compared with placebo (Table 2). Effect sizes were available for ADHD symptom outcomes in 12 of these 14 studies and were 0.5 or above in three of the five studies of stimulants and three of the seven studies of non-stimulants (Table 5). Effect sizes could not be calculated for two studies that reported significant effects of medications on ADHD symptoms [52, 85]. Two studies did not find significant effects of medication on ADHD symptoms: a parallel-group study of reboxetine (reboxetine, *n* = 23; placebo, *n* = 17) [79], and a parallel-group study of dextroamphetamine in patients receiving cognitive behavioural therapy (dextroamphetamine, *n* = 23; placebo, *n* = 25) [96].

**Table 5:**
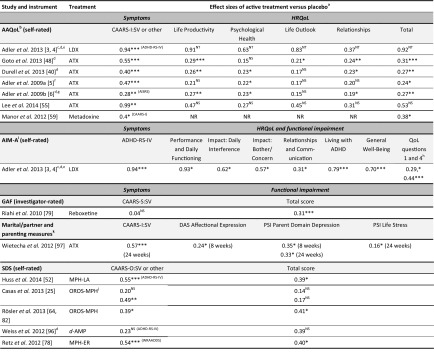
Summary of treatment effect sizes in adults

Effect sizes have been rounded to 2 decimal places. Positive effect sizes indicate a beneficial effect of treatment compared with placebo

*AAQoL* Adult ADHD Quality of Life, *ADHD* attention-deficit/hyperactivity disorder, *ADHD*-*RS*-*IV* ADHD Rating Scale IV [investigator-rated unless otherwise stated], *AIM*-*A* ADHD Impact Module-Adult, *AISRS* Adult ADHD Investigator Symptom Rating Scale, *ATX* atomoxetine, *CAARS*-*I:SV* Conners’ Adult ADHD Rating Scale-Investigator: Screening Version, *CAARS*-*O:SV* Conners’ Adult ADHD Rating Scale-Observer: Screening Version, *CAARS*-*S:SV* Conners’ Adult ADHD Rating Scale-Self-rated: Screening Version, d-*AMP* dextroamphetamine, *DAS* Dyadic Adjustment Scale, *GAF* Global Assessment of Functioning, *HRQoL* health-related quality of life, *LDX* lisdexamfetamine, *MPH*-*ER* methylphenidate extended release, *MPH*-*LA* long-acting methylphenidate, *NR* not reported, *NS* not significant, *NT* not tested, *OROS*-*MPH* osmotic-release oral system methylphenidate, *PSI* Parenting Stress Index, *QoL* quality of life, *SD* standard deviation, *SDS* Sheehan Disability Scale, *WRAADDS* Wender–Reimherr Adult Attention Deficit Disorder Scale

* *p* < 0.05; ** *p* < 0.01; *** *p* < 0.001 (active treatment vs placebo)

^a^Effect sizes could not be calculated for Rösler et al. 2013 [82], the only study to use the Quality of Life Enjoyment and Satisfaction Questionnaire

^b^Effect sizes could not be calculated for Adler et al. 2008 [7], which used the AAQoL, Driving Behavior Survey and Endicott Work Productivity Scale

^c^Patients with ADHD and executive function deficits. Primary efficacy outcome measure: Behaviour Rating Inventory of Executive Function-Adult (ADHD-RS-IV secondary)

^d^Effect sizes of active medication versus placebo were calculated as the difference divided by pooled SD using published data (mean change, n, and SD or standard error of the mean in each group; or n in each group and F-statistic [as appropriate])

^e^Effect sizes were published for ADHD-RS-IV and calculated from the ClinicalTrials.gov study entry for the functional/HRQoL measure (AAQoL)

^f^Patients with comorbid social anxiety disorder

^g^Effect sizes were calculated using values estimated from published bar charts

^h^Effect sizes could not be calculated for AIM-A QoL questions 2 and 4; no significant differences were reported

^i^Effect sizes could not be calculated for Casas et al. 2013 [25] or for Spencer et al. 2008a and b [84, 85]

^j^OROS-MPH doses of 54 mg or 72 mg. Results for each arm are shown

^k^Only significant results are shown; 10 other outcomes were tested and were not significant (see Table 2)

#### HRQoL in adults

Of the 11 studies that assessed HRQoL in adults, significant improvement versus placebo in at least one measure or domain was reported in nine (Table 2). Of these nine, effect sizes were available for six and were 0.5 or above only in a 10-week study of LDX that used the AAQoL [3]. In the studies that used the AAQoL, placebo-adjusted effects varied across the four domains, with the largest effect sizes usually in the Life Productivity domain (0.21–0.91) (Table 5) [3, 5–7, 40, 48, 55]. The other HRQoL measures used in adults were the Quality of Life Enjoyment and Satisfaction Questionnaire (Q-LES-Q) [82] and the Overall Quality of Life (QoL) section of the AIM-A. Significant effects of stimulants versus placebo were reported using the latter measure in two studies, with an effect size of 0.44 for LDX [3, 84, 85].

#### Functional impairment in adults

Of the 10 studies that assessed functional impairment in adults, significant improvements versus placebo in at least one measure were reported in nine (Table 2). Of these nine studies, effect sizes were available for eight and were 0.5 or above in at least one measure or domain in only one study (Table 5) [3]. This revealed significant effects of LDX in all six domains of the AIM-A, with effect sizes of 0.93 in the Performance and Daily Functioning domain and 0.57–0.79 in four of the remaining five domains (*p* < 0.05) (Table 5) [3]. Effect sizes were not calculable for the other two studies that used the AIM-A, but significant placebo-adjusted effects of MAS-XR3 (triple-bead mixed amphetamine salts extended release) were reported in all domains in one study [85] and of OROS-MPH in three to four domains, depending on dose, in the other [25]. Five studies used the self-rated Sheehan Disability Scale (SDS), a global measure of functional impairment across domains of Work/School, Social Life and Family Life. Significant effect sizes of long-acting MPH versus placebo were about 0.4 in three of these studies (Table 5) [25, 78, 82]. Functional impairment was assessed in only three studies of non-stimulants, with significant effects of medication versus placebo in measures of global functioning, marital/family functioning and driving behaviour; effect sizes were about 0.3, when available [7, 79, 97].

### Relationship between ADHD symptom measures and HRQoL/functional measures

To assess the relationship between improvements in ADHD symptoms and improvements in HRQoL or functioning, we collated effect sizes for active treatment versus placebo in all studies, when reported or calculable. Data were available for 16 studies (19 active treatment groups) in children and adolescents and 14 studies in adults (Tables 4, 5).

#### Children and adolescents

Effect sizes were greater for ADHD symptom outcomes than for HRQoL or functional outcomes in all but two studies of children and adolescents; the exceptions were a 2-week study of TD-MPH (effect sizes 0.90 for ADHD-RS-IV and 1.08 for investigator-rated BSFQ) and an 8-week study of ATX (effect sizes 0.33, 0.62 and 0.60 for ADHD-RS-IV total score and 0.47, 0.66 and 0.87 for CHQ-PF50 Psychosocial summary score [low, medium and high dose, respectively]) [66, 99]. Effect sizes were 0.5 or above for both ADHD symptoms and at least one aspect of HRQoL or functioning in five out of 13 non-stimulant treatment groups (ATX or GXR) [42, 66, 86, 91, 94] and five out of six stimulant treatment groups (LDX, OROS-MPH or TD-MPH) [14, 15, 99]. Effect sizes of active medications versus placebo were 0.5 or above for ADHD symptoms but below 0.5 for HRQoL or functioning in five out of 13 non-stimulant treatment groups (ATX or GXR) [17, 37, 51, 68, 101] and one out of six stimulant treatment groups (MPH) [1]. Effect sizes were below 0.5 for both ADHD symptoms and HRQoL or functioning in the remaining three non-stimulant treatment groups (ATX or adjunctive GXR) [51, 65, 100].

Some studies investigated the relationship between ADHD symptom measures and HRQoL or functional measures and consistently identified significant associations. Escobar et al. [42] reported that CHIP-CE:PRF Risk Avoidance domain T-scores correlated moderately with ADHD-RS-IV total scores at baseline (Pearson’s R, −0.46; *p* < 0.05), with weak but significant correlations in the other four domains of the CHIP-CE:PRF and in the Risk Avoidance and Comfort domains of the self-rated CHIP-CE:SRF/AE. In an 8-week GXR study, mean changes from baseline in WFIRS-P total score were significantly greater in patients who responded symptomatically (defined as a CGI-Improvement score of 1 or 2 with ≥30% reduction in ADHD-RS-IV total score) than in those who did not (*p* < 0.001) [86]. Finally, improvement in ADHD-RS-IV total score was significantly correlated with improvement in CHQ-PF-50 scores in a post hoc analysis [62] of an 8-week study of ATX [66].

#### Adults

Effect sizes were greater for ADHD symptom outcomes than for HRQoL or functional outcomes in all but one of the 12 studies (seven of non-stimulants and five of stimulants) that reported significant effects of medication on ADHD symptoms in adults (Table 5). Effect sizes were at least 0.5 for both ADHD symptoms and at least one aspect of HRQoL or functioning in two out of these 12 studies: a 10-week study of LDX that used the AAQoL and AIM-A [3, 4] and a 10-week study of ATX that used the AAQoL (although effects in the latter were not significant) [55]. Effect sizes were at least 0.5 for ADHD symptoms but below 0.5 for HRQoL or functioning in four out of these 12 studies: two of long-acting MPH and two of ATX [48, 52, 78, 97]. Effect sizes were below 0.5 for both ADHD symptoms and HRQoL or functioning in six out of these 12 studies: three of ATX, two of OROS-MPH and one of metadoxine [25, 40, 59, 79, 82, 96].

## Discussion

This systematic review aimed to evaluate HRQoL and functional outcomes in published randomized placebo-controlled clinical trials of medications for ADHD. Tandem extraction of primary or principal symptom-based efficacy outcomes alongside HRQoL and functional outcomes allowed the results of interest to be considered in the context of the effect of study drugs on ADHD symptoms.

Baseline data from the included studies were consistent with published observational studies in showing that children and adolescents with ADHD have substantially impoverished parent-rated HRQoL compared with population norms [32, 80]. Adult HRQoL instruments used in the studies were nearly all ADHD specific, and baseline values are therefore not comparable with reference populations. Baseline deficits on all multi-domain HRQoL and functional impairment instruments, whether generic or ADHD specific, or self- or proxy-rated, were more pronounced in domains relating to achievement/productivity, risk-taking and interpersonal relations than in other psychosocial domains. Little or no impairment was evident in domains relating to physical functioning on the generic instruments. Participants in the included studies were not selected based on HRQoL assessments, but were usually required to have moderate to severe ADHD symptoms (e.g. ADHD-RS-IV total score ≥28), indicating that the poor HRQoL observed at baseline is a reflection of the patients’ characteristics rather than the study inclusion criteria. Overall, these findings support the notion that day-to-day functional impairments and HRQoL deficits are typical in patients with ADHD.

Pharmacotherapy relieved ADHD symptoms to a significantly greater extent than did placebo in all but two of the included studies, as would be expected from the large body of published clinical trials and recent meta-analyses [33, 77, 88, 89]. The two studies in which no significant benefit over placebo was found were both small studies in adults (*N* ≤ 50), one of reboxetine and one of dextroamphetamine. Effect sizes of medication versus placebo for ADHD symptom-based outcomes (when available) were above 0.5 in nearly all of the included studies in children and adolescents, and in most of the studies in adults. We used an effect size of 0.5 as an approximate but universally applicable threshold for a clinically meaningful difference. This is supported by a study showing that nearly all estimates of minimum important differences for HRQoL instruments were close to 0.5 standard deviations and that this may correspond to the limit of people’s ability to discriminate differences over a range of criteria [71].

Functional impairment and poor HRQoL can be viewed as constructs that relate to, but are distinct from, ADHD symptoms and that reflect the impact of ADHD on patients’ day-to-day lives (Fig. 1). In support of this conceptualization, severity of or improvement in ADHD symptoms correlated moderately to strongly (but not perfectly) with severity of or improvement in functional impairment and HRQoL deficits, both in the included studies for which this was reported [42, 62, 66, 86] and elsewhere in the literature [18, 19, 21, 32, 47, 81, 92, 95]. Furthermore, effect sizes (when available) of active medications versus placebo were smaller for functional or HRQoL outcomes than for symptom-based outcomes in nearly all of the included studies (Tables 4, 5). None of this evidence, however, reveals whether medications affect functioning and HRQoL directly, or only through the medium of symptom relief, or both.

In children and adolescents, large effect sizes (≥0.8) were observed for HRQoL and/or functional outcomes in short-term studies of long-acting stimulant medications (LDX, OROS-MPH and TD-MPH). Large effect sizes were not observed in any included studies of non-stimulants (ATX and GXR) and were below 0.5 in many of these. Treatment effect sizes in the included studies suggest that placebo-adjusted improvements in some measures of functioning and of HRQoL are likely to be of sufficient magnitude to be considered potentially clinically relevant, especially with stimulant treatment. Large effect sizes in an individual domain of an instrument may not necessarily reflect large overall effects on HRQoL if some domains have less impact on overall HRQoL than others according to the instrument’s factor structure. Nevertheless, effect sizes on multi-domain instruments were largest in domains relating to achievement/school, risk-taking and interpersonal relations, reflecting the domains with the greatest deficits observed at baseline. In adults, stimulant and non-stimulant effect sizes for functional or HRQoL outcomes were below 0.5 in all included studies except the single adult LDX study. Before drawing any conclusions about the effectiveness of medications or the nature of impairments and deficits in different age groups, however, it should be noted that almost all HRQoL and functional outcomes were parent-rated in child/adolescent studies and self-rated in adult studies. If HRQoL is a subjective experience that can only be judged by patients themselves, then it is questionable whether HRQoL can really be rated by parents, teachers or investigators acting as proxies. Proxy-rated HRQoL assessments may therefore be more appropriately regarded as measures of functional impairment than of HRQoL.

The diversity of instruments used to assess HRQoL and functional impairment in the clinical trials of ADHD medications included in this review suggests a continuing lack of consensus in the field, and perhaps more generally, about which non-symptom-based outcomes should be included and how they should best be assessed. For example, the only HRQoL or functional impairment data identified in this systematic review for patients receiving OROS-MPH were from studies of LDX or ATX in which OROS-MPH was a reference treatment; and the only functional impairment data for patients receiving ATX were from a study of GXR in which ATX was a reference treatment. While some of the differences in choice of HRQoL or functional impairment outcome measure may reflect the changing demands of regulators, it is also the case that the design of a clinical trial involves balancing inclusion of additional outcomes of interest with the feasibility of conducting the study and analysing the results. For example, the use of parent-rated instruments in child/adolescent studies may reflect concerns that some young patients would be unable to complete questionnaires [31, 34], while the use of ADHD-specific HRQoL instruments in adults may reflect concerns that generic instruments would not be sensitive to the impairments characteristic of ADHD in adults. The use of these instruments may also reflect a lack of suitable alternatives: this systematic review focussed only on instruments that have been used in published randomized placebo-controlled studies in patients with ADHD. These complexities reflect the difficulty of sampling the key domains of impairment in individual patients from different perspectives, without merely sampling symptoms. It will be interesting to see whether recent work to develop a standardized nomenclature and toolkit to describe and code functional impairment in people with ADHD based on the World Health Organization’s International Classification of Functioning, Disability and Health will result in a more cohesive and structured approach to assessing HRQoL and functional impairment in clinical trials than at present [35, 36].

This systematic review was limited to randomized, double-blind, placebo-controlled studies published in peer-reviewed journals. The limitations of this approach should be considered when interpreting the results. One potential source of bias is that secondary outcomes or entire studies that do not favour the ADHD medication being tested may be less likely to be published than those that indicate beneficial effects. To limit this bias, we excluded post hoc analyses because they may be more likely than pre-specified analyses to report results favouring the investigational product. Another potential source of bias is that the review did not aim to assess the safety of ADHD medications. Side effects, adverse events or poor tolerability may themselves negatively affect present or future functioning and HRQoL, in addition to other potentially undesirable effects. Finally, the review included studies with different enrolled populations: although all studies enrolled patients with diagnosed ADHD, recruitment criteria differed among studies. For example, some studies included patients with specific psychiatric comorbidities or inadequate responses to previous ADHD medication. This could have affected the qualitative synthesis if study population factors are responsible for differential effects of medication across studies. Despite these limitations, and the limitations of the included studies, the results presented here comprise the most robust available evidence to date that ADHD medications not only provide effective relief of symptoms, but may also reduce functional impairments and improve HRQoL in children, adolescents and adults with ADHD. Furthermore, real-world evidence from registry studies suggests that this may be the case for patients in clinical practice [16, 56, 57], as well as for those enrolled in clinical trials.

Whether pharmacological therapy is appropriate, and if so which of the treatment options and which accompanying behavioural or psychological intervention is most suitable for each patient, are decisions for healthcare professionals, parents and patients. The evidence presented here should encourage everyone involved in a patient’s treatment to aim for reduced functional impairment and improved HRQoL, as well as relief of symptoms, for all patients with ADHD.

## Electronic supplementary material

Below is the link to the electronic supplementary material.

Supplementary material 1 (DOCX 42 kb)
