# Supplementary material for: Systematic review of quality of life and functional outcomes in randomized placebo-controlled studies of medications for attention-deficit/hyperactivity disorder
Source: Eur Child Adolesc Psychiatry. 2017 Apr 20;26(11):1283–307. doi: 10.1007/s00787-017-0986-y (PMC5656703; doi:10.1007/s00787-017-0986-y)
Supplement: Supplementary file 1 — Supplementary material 1 (DOCX 42 kb) [file 787_2017_986_MOESM1_ESM.docx]

#### Supplementary Table 1 PubMed search string

|  | **Search string** |
| --- | --- |
| **Language**  **Randomized, placebo-controlled studies**  **Patients with ADHD**  **(HR)QoL/functional outcome** | english[la]  **AND**  ((placebo*[tiab] OR PBO[tiab] OR placebos[mh])  AND  (random*[tiab] OR randomized controlled trial[pt] OR randomized controlled trials as topic[mh] or random allocation[mh])  NOT  (animals[mh] NOT humans[mh])  NOT  (letter[pt] OR historical article[pt] OR case study[tiab] OR case report[tiab]))  **AND**  (attention deficit disorder with hyperactivity[mh] OR (attention[tiab] AND deficit[tiab] AND disorder[tiab]) OR ADHD[tiab] OR AD/HD[tiab] OR (hyperkinetic[tiab] AND disorder[tiab]))  **AND**  ((quality of life[mh] OR HRQoL[tiab] OR HRQL[tiab] OR QoL[tiab] OR quality of life[tiab])  OR  (function*[tiab] NOT (magnetic resonance imaging[mh] OR (magnetic[tiab] AND resonance[tiab]) OR mri[tiab] OR fmri[tiab]))  OR  (WFIRS*[tiab] OR CHIP*[tiab] OR (child health and illness profile*[tiab]) OR AIM-A[tiab] OR AIM-C[tiab] OR (impact module*[tiab]) OR (EQ-5D*[tiab]) OR Q-LES-Q[tiab] OR AAQoL[tiab] OR CHQ[tiab] OR (child health questionnaire*[tiab]) OR PEDSQL[tiab] OR YQOL[tiab] OR QOLI[tiab] OR GAF[tiab] OR Sheehan disability scale[tiab] OR SDS[tiab] OR SDQ[tiab] OR strengths and difficulties questionnaire[tiab] OR KINDL[tiab] or BADDS[tiab])) |

*ADHD* attention-deficit/hyperactivity disorder, *HRQoL* health-related quality of life, *[la]* language, *[tiab]* title and abstract, *[mh]* medical subject heading
